# Micro- and macrovascular function in patients suffering from primary adrenal insufficiency: a cross-sectional case–control study

**DOI:** 10.1007/s40618-020-01309-2

**Published:** 2020-06-01

**Authors:** M. Müller, H. Beiglböck, P. Fellinger, Y. Winhofer, A. Luger, M. Gschwandtner, A. Willfort-Ehringer, R. Koppensteiner, A. Kautzky-Willer, M. Krebs, O. Schlager, P. Wolf

**Affiliations:** 1 Division of Angiology, Department of Internal Medicine II, Medical University of Vienna, Vienna, Austria; 2 Division of Endocrinology and Metabolism, Department of Internal Medicine III, Medical University of Vienna, Währinger Gürtel 18-20, 1090 Vienna, Austria

**Keywords:** Glucocorticoid replacement therapy, Mineralocorticoid replacement therapy, Addison’s disease, Renin–angiotensin–aldosterone system

## Abstract

**Background:**

Despite adequate glucocorticoid (GC) and mineralocorticoid (MC) replacement therapy, patients suffering from primary adrenal insufficiency (AI) have an increased mortality, mainly due to cardiovascular diseases. Only little knowledge exists on the contribution of MC substitution to the cardiovascular risk. Therefore, this study investigates the impact of plasma renin concentration on parameters of micro- and macrovascular function.

**Methods:**

26 patients with primary AI [female = 18, age: 51 (28; 78) years; BMI: 24 (18; 40) kg/m^2^; disease duration: 18 (5; 36) years] were included in this cross-sectional analysis. Intima media thickness (IMT) and pulse wave velocity (PWV) were investigated to assess macrovascular remodeling and arterial stiffness. Microvascular function was estimated by post-occlusive reactive hyperemia using laser Doppler fluxmetry. Baseline perfusion, biological zero, peak perfusion, time to peak and recovery time were recorded. Patients were grouped according to their median plasma renin concentration of previous visits (Renin_high_ vs Renin_low_) and were compared to a group of healthy women [age: 44 (43; 46) years; BMI: 24.2 (21.8; 27.5)].

**Results:**

PWV was significantly higher in AI patients compared to controls [9.9 (5; 18.5) vs 7.3 (6.8; 7.7) m/s; *p* < .01], whereas no differences in microvascular function could be found. In Renin_low_ time to peak perfusion was significantly longer [6.0 (3; 15) vs 3.5 (1.5; 11) s; *p* < .05], whereas no differences in IMT and PWV were observed between Renin_high_ and Renin_low_. No impact of GC dose was observed.

**Conclusions:**

Microvascular function is not impaired in patients with primary AI under adequate replacement therapy, although higher renin concentrations are associated with subclinical improvements. No relation between RAAS activity and macrovascular function is observed, while arterial stiffness might be increased in primary AI.

## Background

Primary adrenal insufficiency (AI) is a rare disease, most commonly caused by autoimmunity in industrialized countries [1]. Despite adequate glucocorticoid (GC) and mineralocorticoid (MC) replacement therapy, evidence suggests that AI might be associated with an increased mortality, mainly due to cardiovascular (CV) diseases [2]. GC and MC both play an important role in the CV system and are well known to affect CV risk factors in general population [3].

With regard to GC replacement therapy, even a small oversupply in daily hormone substitution is associated with an increase in waist circumference, blood pressure and fasting glucose [4]. Moreover, short-term increase in daily GC dose for 1 week results in endothelial dysfunction in patients with secondary AI [5]. Also unphysiological timing might play a role, as modified release formulations of GC improve body weight, glucose and lipid metabolism compared to conventional substitution therapy, independently of total daily GC dose [6, 7].

Only little knowledge exists on the impact of MC replacement therapy in the development of CV risk factors. We have recently shown that primary AI is characterized by a unique profile of different angiotensin metabolites and that the renin–angiotensin–aldosterone system (RAAS) is highly upregulated despite adequate hormone replacement therapy [8]. In addition, higher renin concentrations are associated with a more favorable cardiac function and morphology in patients with primary AI [9].

Aldosterone secretion is regulated by the RAAS, which consists of an enzymatic cascade with numerous angiotensin metabolites. Whereas the adverse effects of excessive aldosterone secretion on vasculature, endothelial function and myocardial hypertrophy are well known [10, 11], evidence for the role of other angiotensin metabolites of the RAAS is scarce. The most prominent metabolite is Angiotensin 2, which contributes to the development of CV diseases [12], whereas other metabolites might exert protective counter-regulatory effects [13]. We have previously shown that RAAS activity closely correlates with plasma renin concentrations in patients with primary AI [8], but the effects on vascular function are not known.

Microvascular changes, such as endothelial dysfunction and arterial rarefaction, occur early in a variety of pathological conditions linked with cardiovascular disease [14]. Microvascular function in human skin has been suggested as an easily accessible surrogate for the assessment of respective changes in other vascular beds, such as the coronary microcirculation. Accordingly, impaired endothelium-dependent vasodilation and capillary recruitment in skin have been shown to correlate with an increased risk for coronary heart disease [15]. Laser Doppler fluxmetry (LDF) is a noninvasive means for continuous assessment of microvascular perfusion, based on measurement of the Doppler shift that results from coherent monochromatic light scattering by moving erythrocytes [16]. When studying microvascular reactivity, LDF is commonly applied in conjunction with provocative stimuli, such as artery occlusion in the context of post-occlusive reactive hyperemia (PORH), characterized by peak perfusion and sustained hyperemia [17]. Laser-Doppler-derived parameters such as peak perfusion and time to peak have shown good reproducibility in the assessment of microvascular function [18].

Ultrasound examination of extracranial cerebral arteries allows for assessment of carotid plaque burden, predictive of cardiovascular events [19], and estimation of intima media thickness (IMT), a standard marker of atherosclerosis [20].

Arterial stiffening, related to damage in the arterial wall, can be assessed by measuring aortic pulse wave velocity (PWV). Arterial stiffness predicts future cardiovascular disease and can be used for risk classification [21].

Therefore, this study aims to investigate the impact of plasma renin concentration, as well as daily GC replacement dose on parameters of micro- and macrovascular remodeling in patients with primary AI under stable hormone replacement therapy.

## Methods

A cross-sectional, single-center case–control study was performed at the Division of Endocrinology and Metabolism, Department of Internal Medicine III in cooperation with the Division of Angiology, Department of Internal Medicine II of the Medical University of Vienna.

The protocol was approved by the local ethical committee of the Medical University of Vienna and written informed consent was obtained from all participating subjects. This study was conducted in conformance with the relevant guidelines and regulations, i.e., principles of the Declaration of Helsinki and the ICH-GCP guidelines.

Patients with confirmed primary AI in regular care at the endocrine outpatient clinic at the Medical University of Vienna were searched for available plasma renin concentrations of previous visits. Included patients had to be on stable GC and MC replacement therapy for at least 6 months previous to all study-related activities. Exclusion criteria were concomitant antihypertensive therapy with ACE inhibitors/AT2 antagonists, diabetes mellitus (type 1 and 2), elevated liver enzymes > 3 × upper limit of normal, medical history of CV disease and advanced chronic kidney disease (estimated glomerular filtration rate < 45 ml/min). Furthermore, no patient was under therapy with diuretics or aldosterone receptor antagonists.

26 patients with biochemically proven primary AI due to autoimmune adrenalitis (*n* = 24) or adrenoleukodystrophy (*n* = 2) were included in the study. In all patients, GC replacement therapy consisted of standard rapid release hydrocortisone divided in two doses, taken immediately in the morning and in the afternoon. All patients included in analysis received daily MC replacement therapy, taken once daily in the morning.

The control group consisted of 27 healthy female volunteers without a history of disturbed glucose tolerance, severe obesity, hypertension and cardiovascular diseases. Data of the control group were in parts published previously [22]. Of note, subjects in this historical control group were significantly younger than patients with primary AI. Therefore, additional comparisons of parameters of macro- and microvascular function were performed in a group of 16 patients with primary AI ≤ 55 years, matched for age [47 (28; 55) vs 44 (43; 46) years], as well as in female AI patients only (*n* = 18) matched for sex.

All subjects underwent clinical examination, blood tests, the assessment of anthropometric characteristics and measurements of macro- and microangiopathy.

To investigate the impact of RAAS activity, patients were grouped according to their median plasma renin concentrations at previous visits [3.9 (2; 4) visits] at the endocrine outpatient clinic (Renin_low_: < 100 μIU/ml and Renin_high_: > 100 μIU/ml = twofold upper normal range).

To evaluate the impact of daily GC replacement therapy, patients were divided into daily hydrocortisone dose of ≤ 12 mg/day/m^2^ body surface area (HC_low_) and daily hydrocortisone dose of > 12 mg/day/m^2^ body surface area (HC_high_), according to recent clinical practice recommendations [23].

Blood tests were performed under standardized conditions in seated position following at least 15 min of physical rest. Laboratory parameters were measured by routine lab methods at the Department of Laboratory Medicine of the Medical University of Vienna (https://www.kimcl.at). Blood was drawn in the fasting state about 12–16 h after the last intake of regular daily hormone substitution.

Micro- and macroangiopathy was assessed by laser Doppler fluxmetry (LDF) and measurement of PWV as well as carotid ultrasound with determination of intima media thickness (IMT), respectively. LDF examination was performed with the patient in supine position in a quiet room at ambient temperature after an acclimatization period of 5 min. The moorVMS-LDF2 Laser Doppler monitor (Moor Instruments, Devon, United Kingdom) and respective software (moorVMS-PC V3.1) were used for all LDF measurements. Probes were positioned at the volar site of the distal forearm, after local disinfection. Brachial artery occlusion during post-occlusive reactive hyperemia (PORH) measurement was performed by suprasystolic cuff inflation proximal to the cubita. At first baseline flux was recorded for 3 min. After cuff inflation, biological zero flux was recorded for another 3 min until cuff deflation and consecutive measurement of reactive hyperemia flux. LDF-derived parameters were baseline flux, biological zero, peak perfusion, time to peak, recovery time, percent change from baseline perfusion to peak perfusion, and percent change from biological zero to peak perfusion.

LDF measurements in the control group had been performed using PIM II laser Doppler perfusion measurement system (with LISCA Opto-Isolation Unit; Perimed, Järfälla, Sweden) and details on the measurement setup have been published previously [22]. To account for potential confounders due to different instruments we compared the relative changes of perfusion between the groups.

Consecutively, PWV was measured with the patient still in supine position. The AngE Angio Experience device (Sonotechnik Austria) and respective PC-Software were used for all measurements. Electrodes for ECG-gating were placed on both upper extremities. Cuffs were positioned at the distal forearms and lower legs and their respective distances from the mid-portion of the sternum were measured in cm. PWV was measured in m/s.

Bilateral carotid ultrasound was performed to assess for carotid plaque burden and IMT. An institutional standard ultrasound protocol was used for assessment of the common carotid, external and internal carotid arteries as well as vertebral arteries and IMT measurement.

Exploratory statistical analysis was performed using SPSS Version 24 (IBM, Armonk, NY, USA). Data are given as median (minimum; maximum) or inter quartile range. Comparison between groups was performed by Mann–Whitney *U* tests. Correlation analysis was calculated with Spearman’s correlation coefficient (*r*). Multivariable linear regression analysis was performed using R Version 3.6.1 (R Core Team (2016). R: A language and environment for statistical computing. R Foundation for Statistical Computing, Vienna, Austria.). Due to the hypothesis-generating design of the study data were not corrected for multiple testing. Level of statistical significance was set at *α* < 0.05.

## Results

Twenty-six patients (female = 18) participated in the study. Anthropometrical and clinical characteristics did not differ between Renin_low_ vs Renin_high_ and HC_low_ vs HC_high_. No differences were found in parameters of glucose and lipid metabolism. Also median plasma renin concentration did not differ between HC_low_ vs HC_high_. With regard to parameters of systemic inflammation, interleukin-6 concentration was significantly higher in Renin_high,_ whereas CRP levels were comparable. No differences in inflammatory markers were observed in HC_low_ compared to HC_high_. Data are given in detail in Table 1.

**Table 1:** Anthropometric and clinical characteristics of all included patients suffering from primary AI, as well as of patients grouped according to their median renin levels in previous visits and their daily dose of glucocorticoid replacement therapy

	Total	Renin_low_	Renin_high_	HC_low_	HC_high_
*n*	26	11	15	19	7
Age (years)	51 (28; 78)	55 (29; 75)	51 (28; 78)	55 (28; 78)	51 (29; 60)
BMI (kg/m^2^)	24 (18; 40)	25 (19; 35)	24 (18; 40)	25 (18; 40)	25 (19; 40)
Disease Duration (years)	18 (5; 36)	18 (10; 35)	17 (5; 36)	18 (5; 35)	20 (7; 36)
HC dose (mg/day/m^2^ BSA)	11.4 (9.5; 19.5)	11.4 (9.5; 19.5)	11.5 (9.5; 17.9)	11 (9.5; 12.5)	17.3 (13.7; 19.5)*
MC dose (mg/day)	0.05 (0.01; 0.11)	0.05 (0.01; 0.1)	0.07 (0.02; 0.11)	0.05 (0.01; 0.1)	0.06 (0.05; 0.11)
Systolic RR (mmHg)	129 (100; 166)	129 (100; 139)	121 (95; 145)	123 (105; 166)	122 (95; 134)
Diastolic RR (mmHg)	73 (58; 93)	73 (67; 84)	84 (60; 93)	73 (58; 94)	83 (66; 93)
Median renin (μIU/ml)	118 (4; 1501)	46 (4; 97)	201 (101; 1501)*	101 (19; 717)	97 (169 (4; 1501)
Sodium (mmol/l)	138 (130; 145)	139 (133; 145)	138 (130; 141)	139 (133; 145)	137 (130; 139)
Potassium (mmol/l)	4.3 (3.8; 5.3)	4.3 (3.8; 5.1)	4.4 (3.8; 5.3)	4.3 (3.8; 5.2)	4.2 (3.9; 5.3)
Creatinine (mg/dl)	0.9 (0.7; 1.2)	0.89 (0.8; 1.1)	0.9 (0.7; 1.2)	0.9 (0.7; 1.1)	0.9 (0.7; 1.2)
Triglycerides (mg/dl)	105 (44; 288)	70 (44; 222)	124 (77; 247)	105 (44; 222)	107 (59; 247)
Cholesterol (mg/dl)	191 (38; 247)	167 (145; 245)	194 (159; 247)	183 (145; 251)	192 (156; 247)
HDL Cholesterol (mg/dl)	59 (38; 89)	59 (43; 89)	59 (38; 89)	59 (38; 89)	54 (48; 83)
LDL Cholesterol (mg/dl)	101 (43; 186)	87 (64; 186)	114 (43; 147)	92 (43; 186)	124 (64; 147)
Glucose (mg/dl)	82 (68; 106)	82 (77; 106)	83 (68; 96)	82 (68; 106)	82 (77; 92)
HbA1c (%)	5.2 (4.7; 5.8)	5.2 (4.7; 5.7)	5.4 (4.8; 5.8)	5.2 (4.7; 5.7)	5.5 (5.9; 5.8)
HOMA	2.1 (0.9; 6.5)	1.9 (0.9; 6.5)	2.3 (0.9; 5.1)	2.0 (0.9; 6.5)	2.4 (0.9; 5.1)
CRP (mg/dl)	0.11 (0.03; 0.5)	0.06 (0,03; 0.5)	0.15 (0.03; 0.3)	0.11 (0.03; 0.5)	0.13 (0.03; 0.3)
Il-6 (pg/ml)	3.1 (1.5; 7.9)	2.5 (1.5; 5.4)	4.8 (1.5; 7.9)*	3.8 (1.5; 7.9)	2.7 (1.5; 7.02)

*BMI* body mass index, *HC* hydrocortisone, *MC* mineralocorticoid, *BSA* body surface area, *systolic and diastolic RR* mean systolic and diastolic blood pressure in previous visits at the outpatients clinic, *Il-6* interleukin 6

*p < 0.05

When patients were investigated according to their RAAS activity in previous visits, no differences in parameters of macroangiopathy and arterial stiffness, i.e., IMT and PWV, were found. Analysis of surrogate parameters of microangiopathy showed a significantly longer time to peak perfusion in Renin_low_, whereas baseline perfusion, biological zero, peak perfusion and recovery time were similar between the groups (see Table 2). However, there was no significant association between median renin levels and parameters of micro- and macroangiopathy in correlation analysis.

**Table 2:** Differences in surrogate parameters of macroangiopathy (intima media thickness), arterial stiffness (pulse wave velocity) as well as microangiopathy (baseline perfusion, biological zero, peak perfusion, time to peak perfusion and recovery time) in patients with primary AI according to median plasma renin concentrations at previous visits; Renin_low_: median renin levels < 100 μIU/ml; Renin_high_: median renin levels > 100 μIU/ml

	Renin_low_ (*n* = 11)	Renin_high_ (*n* = 15)	*p* value
Intima media thickness (mm)	0.64 (0.4; 1.2)	0.62 (0.4; 0.1)	0.31
Pulse wave velocity (m/s)	10.2 (6.5; 18.5)	9.8 (5.1; 14.2)	0.97
Baseline perfusion (AU)	10.6 (5.3; 20.4)	9.9 (4.8; 24.1)	0.57
Biological zero (AU)	3.2 (2.2; 7.3)	3.1 (1.4; 5)	0.36
Peak perfusion (AU)	54.0 (25; 73)	55 (14; 110)	0.73
Time to peak perfusion (s)	6.0 (3; 15)	3.5 (1.5; 11)	< 0.05*
Recovery time (AU)	93 (25; 180)	78 (14; 180)	0.91

*indicates *p* < 0.05

No differences in markers of macro- and microangiopathy were observed between HC_low_ vs HC_high_ (see Table 3).

**Table 3:** Differences in surrogate parameters of macroangiopathy (intima media thickness), arterial stiffness (pulse wave velocity) as well as microangiopathy (baseline perfusion, biological zero, peak perfusion, time to peak perfusion and recovery time) in patients with primary AI according to daily dose of GC replacement therapy; HC_low_: ≤ 12 mg/day/m^2^ body surface area; HC_high:_ > 12 mg/day/m^2^ body surface area

	HC_low_ (*n* = 19)	HC_high_ (*n* = 7)	*p* value
Intima media thickness (mm)	0.64 (0.4; 1.2)	0.59 (0.5; 0.7)	0.32
Pulse wave velocity (m/s)	10.2 (5.1; 18.5)	9.8 (7.1; 12.3)	0.66
Baseline perfusion (AU)	10.0 (4.8; 24)	10.3 (5.2; 17.4)	0.80
Biological zero (AU)	3.1 (1.4; 7.3)	3.2 (2.2; 5)	0.66
Peak perfusion (AU)	54 (14; 96)	55 (41; 110)	0.435
Time to peak perfusion (s)	6 (2; 15)	3.6 (1.5; 14)	0.26
Recovery time (AU)	106 (14; 180)	73 (37; 177)	0.28

In multivariable linear regression analysis PWV was associated with HbA1c, but neither with median renin concentrations, nor daily GC replacement dose (see Table 4).

**Table 4:** Multivariable linear regression model with pulse wave velocity as a dependent variable and plasma renin, hydrocortisone dose, serum-creatinine, LDL-cholesterol, HbA1c%, and age as explanatory variables

	Estimate	Std. error	*p* value
Plasma renin	− 0.005	0.002	0.054
Hydrocortisone dose	− 0.331	0305	0.302
Creatinine	6.167	4.957	0.239
LDL-C	− 0.006	0.023	0.779
HbA1c	9.729	3.411	< 0.05*
Age	− 0.058	0.077	0.464

Adjusted R-squared: 0.44

*indicates *p* < 0.05

Interleukin-6 concentrations positively correlated with median plasma renin levels (*r*: 0.424; *p* = 0.03). No association was found between concentrations of renin and CRP.

In Renin_low_ salt craving was reported by 1 patient (9%), whereas it was present in 6 patients (43%) in Renin_high_. No differences were observed with regard to postural hypotension.

No difference in parameters of macro- and microvascular function was observed between female and male patients with primary AI.

In comparison to the control group of healthy women PWV was significantly higher in patients with primary AI, but no differences in parameters of microvascular function were observed (see Table 5). The observed difference in PWV between the groups was still present in the age-matched subpopulation of AI patients [PWV in patients vs controls: 9.3 (7.9; 10.2) vs 7.3 (6.8; 7.7), *p* < 0.01], as well as when only female patients were analyzed [PWV in female patients vs controls: 9.7 (8.4; 11.2) vs 7.3 (6.8; 7.7), *p* < 0.01].

**Table 5:** Differences in arterial stiffness (pulse wave velocity; PWV) as well as microangiopathy (change from baseline perfusion to peak perfusion, change from biological zero to peak perfusion) in patients compared to healthy controls

	Patients	Controls	*p* value
Age (years)	51 (46; 68)	44 (43; 46)	< 0.01*
Systolic RR (mmHg)	121 (112; 134)	119 (107; 128)	0.39
Diastolic RR (mmHg)	80 (74; 87)	75 (70; 80)	0.15
PWV (m/s)	9.6 (8.3; 10.8)	7.3 (6.8; 7.7)	< 0.01*
Change from baseline to peak perfusion	3.6 (2.6; 5.4)	3.95 (3.3; 4.3)	0.85
Change from biological zero to peak perfusion	13.3 (10.8; 20.7)	11.5 (9.4; 15.6)	0.11

*indicates *p* < 0.05

## Discussion

Our data demonstrate that altered RAAS activity due to AI is not associated with clinically relevant microangiopathy. However, time to peak perfusion in PORH experiments was significantly longer in the Renin_low_ group, probably reflecting subtle subclinical changes in microvascular function. In addition, arterial stiffness is higher in patients suffering from primary AI compared to healthy controls.

In line with that, our recent data [9] indicate that lower plasma renin concentrations are associated with an unfavorable cardiac morphology and function, characterized by a reduced ejection fraction and an increased interventricular septal thickness. Thus, increased RAAS activity might play a role in the observed increase in CV diseases in patients with primary AI.

Most studies addressing CV mortality mainly focus on the importance of daily dose and adequate timing of GC replacement therapy, whereas evidence on the role of MC replacement therapy is scarce. We recently demonstrated that despite adequate, stable, guideline conforms GC and MC substitution therapy the RAAS is highly activated in primary AI [8]. Aldosterone is the most important player of the RAAS and correlates negatively with plasma renin [11]. Supraphysiological concentrations of aldosterone are related to arterial stiffness, impaired vascular compliance and relaxation, but also increased intravascular volume because of renal sodium retention [24]. However, other angiotensin metabolites are as well known to play an active role in modulating CV risk factors. The most prominent metabolite is angiotensin 2, which strongly correlates with plasma renin levels in primary AI [8]. Similar to aldosterone, angiotensin 2 induces vascular injury and promotes atherosclerosis by the production of reactive oxygen species. In addition, angiotensin 2 is a potent proinflammatory agent, which might explain the increased interleukin-6 concentrations in the Renin_high_ group observed in our study [10]. On the other hand, protective angiotensin metabolites in the RAAS cascade, like angiotensin 1–7 and angiotensin 1–5, are significantly upregulated as well [8] and therefore might counteract the adverse CV effects [25].

Of note, our results should therefore be considered in face of recently published clinical practice guidelines, who recommend to titrate fludrocortisone dose to target for plasma renin concentrations in the upper limit of normal [26, 27]. In our studies patients with median renin levels two times higher the upper limit of normal show not only subtle improvements in microvascular function, but also a favorable cardiac function and morphology [9].

On the other hand, frequently observed symptoms of inadequate MC replacement therapy, like salt craving and postural hypotension, might contribute to the reported impairment in quality of life in patients with primary AI [28]. Also in our study, salt craving was much more frequently observed in the Renin_high_ group.

Interestingly, daily fludrocortisone dose was not different between Renin_high_ and Renin_low_, highlighting the importance of other factors besides MC replacement dose in the modulation of RAAS activity, including posture, daytime, dietary sodium intake, volume status and plasma potassium levels [29]. This is in line with a recently published study by Pofi et al., in which modifications in MC dose were not associated with changes in plasma renin concentrations in a large collective of patients with primary AI [30]. Dose adjustments of MC replacement therapy might therefore rather be based on clinical characteristics, including blood pressure, salt craving and postural hypotension.

While the clinical usefulness of plasma renin concentrations for adjustments of MC dose seems to be questionable, we have previously shown that also in patients with AI plasma renin concentrations are closely associated with the activity of the RAAS and plasma angiotensin 2 and are not acutely affected by glucocorticoid and MC intake in patients with primary AI [8]. Therefore, plasma renin concentration is a valid surrogate parameter for RAAS activation.

The major limitation of our study is that the control group is not matched for sex and age. Of note, we did not observe clinically relevant differences in parameters of microangiopathy in patients with long standing primary AI, even when compared to a group of younger women. PWV is higher in primary AI, which might be biased by sex and age, since PWV is well known to increase with age [31]. Also sex hormone status due to oral contraceptives might have an impact, for which we did not to account in our analysis. However, in sex-specific analysis no differences in parameters of macro- and microvascular function between men and women could be found. In addition, the difference in PWV remains almost identical within a subgroup of patients matched for age to the control group.

Another limitation is the intra-individual variability of plasma renin concentrations, mainly depending on dietary salt and water intake, which was not systematically assessed during the experiments. Also the time-period between blood draw and last intake of daily hormone substitution therapy was not standardized. To address this issue we used median renin concentrations of previous visits at the outpatients’ clinic for analysis.

Taken together, our results demonstrate an association between subclinical improvements in microvascular function in patients with primary AI and higher renin concentrations, although no differences compared to healthy controls were observed. No relation between the RAAS and macrovascular function is observed, while arterial stiffness might be increased in primary AI.
